# Qualitative and quantitative assessment of non-clear cell renal cell carcinoma using contrast-enhanced ultrasound

**DOI:** 10.1186/s12894-024-01514-8

**Published:** 2024-06-17

**Authors:** WeiPing Zhang, JingLing Wang, Li Chen, Jiayu Shi

**Affiliations:** 1 Department of Ultrasound, The First Affiliated Hospital, Jiangxi Medical College, Nanchang University, Nanchang, China; 2 The First Clinical Medical College, Jiangxi Medical College, Nanchang University, Nanchang, China

**Keywords:** Contrast-enhanced ultrasound, Qualitative, Non-clear cell renal cell carcinoma, Quantitative

## Abstract

**Background:**

Non-clear cell renal cell carcinoma (nccRCC) represents a rare form of renal cell carcinoma (RCC) in the clinic. It is now understood that contrast-enhanced ultrasound (CEUS) exhibits diverse manifestations and can be prone to misdiagnosis. Therefore, summarizing the distinctive features of contrast-enhanced ultrasonography is essential for differentiation from ccRCC.

**Objective:**

This study aims to evaluate the diagnostic efficacy of qualitative and quantitative CEUS in diagnosing nccRCC to enhance our understanding of this condition.

**Methods:**

We conducted a retrospective analysis of 21 patients with confirmed nccRCC following surgery and assessed the characteristic conventional ultrasound and CEUS imaging features. The paired Wilcoxon signed-rank sum test was employed to compare differences in CEUS time-intensity curve (TIC) parameters between the lesions and the normal renal cortex.

**Results:**

Routine ultrasound revealed the following primary characteristics in the 21 nccRCC cases: hypoechoic appearance (10/21, 47.6%), absence of liquefaction (18/21, 66.7%), regular shape (19/21, 90.5%), clear boundaries (21/21, 100%), and absence of calcification (17/21, 81%). Color Doppler flow imaging (CDFI) indicated a low blood flow signal (only 1 case of grade III). Qualitative CEUS analysis demonstrated that nccRCC predominantly exhibited slow progression (76.1%), fast washout (57%), uniformity (61.9%), low enhancement (71.5%), and ring enhancement (61.9%). Quantitative CEUS analysis revealed that parameters such as PE, WiAUC, mTTI, WiR, WiPI, WoAUC, WiWoAUC, and WOR in the lesions were significantly lower than those in the normal renal cortex (*Z*=-3.980, -3.563, -2.427, -3.389, -3.980, -3.493, -3.528, -2.763, *P* < 0.001, < 0.001, = 0.015, = 0.001, < 0.001, < 0.001, < 0.001, = 0.006). However, there were no significant differences in RT, TTP, FT, or QOF (all *P* > 0.05).

**Conclusion:**

nccRCC exhibits distinctive CEUS characteristics, including slow progression, fast washout, low homogeneity enhancement, and ring enhancement, which can aid in distinguishing nccRCC from ccRCC.

## Introduction

Renal cell carcinoma (RCC) stands as one of the most prevalent malignancies in the field of urology. Approximately 70% of RCC cases manifest as clear cell renal cell carcinoma (ccRCC). In addition to ccRCC, there exist several rarer variants of non-clear cell renal cell carcinoma (nccRCC), including papillary renal cell carcinoma (pRCC), chromophobe renal cell carcinoma (ChRCC), translocation cancer, and collecting duct cancer [1, 2].

The majority of RCC cases remain asymptomatic, frequently detected incidentally through imaging procedures [3]. While enhanced computed tomography (CT) has traditionally served as the gold standard for diagnosing renal cell carcinoma, it carries inherent drawbacks, such as ionizing radiation exposure and the potential for hypersensitivity reactions to iodine-based contrast agents, thereby limiting its application [4]. In contrast, ultrasound, a non-invasive examination, can provide an initial assessment of tumor location, size, shape, and vascularization, making it the preferred method for initial diagnosis [5]. However, conventional ultrasound often exhibits limitations stemming from restricted two-dimensional resolution and discrepancies in tumor images, significantly impeding its effectiveness in diagnosis, differentiation, prognosis prediction, and evaluating the therapeutic outcomes of RCC.

Contrast-enhanced ultrasound (CEUS) has emerged as a more sensitive alternative to conventional ultrasound. It offers clear visualization of microvascular changes within and around tumors, coupled with quantitative analysis software that generates time-intensity curves for qualitative and quantitative assessments of blood perfusion within the lesion. CEUS brings several advantages, including convenience, affordability, broad applicability, absence of ionizing radiation, negligible risk of iodine contrast agent allergies, and the ability to conduct repeated examinations in quick succession. Notably, CEUS demonstrates diagnostic sensitivity and specificity for RCC akin to that of CT [6, 7]. Hence, it has attracted significant interest from clinicians in recent years. ccRCC, characterized by a rich blood supply and susceptibility to hemorrhage, cystic degeneration, and necrosis, typically exhibits a CEUS pattern of “rapid enhancement followed by rapid washout” [8]. Conversely, the histological characteristics of nccRCC are complex and changeable, and the manifestations of different types of tumors may be different in CEUS. It leads to nccRCC presents diverse ultrasound and CEUS manifestations, which often pose a diagnostic challenge prior to surgical intervention [9]. Herein, we retrospectively analyzed the CEUS characteristics of 21 surgically and pathologically confirmed nccRCC cases. Importantly, we sought to enhance diagnostic ability for this tumor subtype and offer valuable guidance for clinical decision-making.

## Materials and methods

### Study patients

From January 2020 to May 2023, 21 patients with nccRCC confirmed by pathology underwent CEUS in the First Affiliated Hospital of Nanchang University. Patients were included in the present study based on the following criteria: (1) Evidence of renal masses on gray-scale ultrasound with complete conventional ultrasound and CEUS imaging data; (2) No history of renal invasive procedures or other treatments before the ultrasound examination; (3) all patients underwent surgical treatment, and pathologically confirmed as nccRCC; (4) age ≥ 18 years; (5) lesion diameter more than 1 cm; (6) Quality of fit (QOF) > 0.7. Exclusion criteria: (1) nccRCC without enhancement or nodular enhancement of the cyst wall; (2) excluding tumors with poor dynamic image storage and large respiratory range, which could not be quantitatively analyzed in the later stage; (3) CEUS images were uninterpretable due to factors such as the deep location of the tumor, patient obesity, or inadequate ultrasound penetration. The study was approved by the hospital’s ethics committee and the institutional review board (NO: IIT2023174), and informed consent was obtained from every participant.

### Instruments

Routine ultrasound and CEUS examinations were conducted using either the *Mindray Resona R9* ultrasonic diagnostic instrument (Shenzhen Mairui Biomedical Electronics Co., Ltd, probe model SC5-1U) or the *Siemens ACUSON Sequoia* ultrasonic diagnostic instrument (Siemens Medical System Co., Ltd, probe model C5-1). Conventional ultrasound was used for the observation of lesion size, location, shape, boundary, internal echo, and blood flow. Subsequently, the optimal section that offered clear visualization of the tumor and surrounding renal parenchyma was selected for CEUS, and the target focus was located in the middle of the image as far as possible. Patients were instructed to maintain calm and slow breathing during the procedure. CEUS was performed using a low mechanical index (MI 0.06–0.08) and SonoVue (SonoVue, 2.5 μm in diameter, Bracco, Italy) contrast medium. Prior to examination, 59 mg of SonoVue was mixed with 5 ml of 0.9% sodium chloride solution to form a suspension. During the imaging procedure, 1.0 ml of the SonoVue suspension was injected through the superficial vein of the elbow, followed by the injection of 5 ml of 0.9% sodium chloride solution to maintain consistency. Dynamic images were captured between 120 s and 180 s. Two experienced ultrasound physicians with over 5 years of expertise in CEUS, blinded to the pathological results, conducted qualitative and quantitative analyses of the images.

### Qualitative analysis of CEUS images

The renal CEUS phase was divided into the perfusion phase (0–30 s) and the washout phase (> 30 s). During this analysis, attention was given to enhancement and washout times, peak intensity, enhancement uniformity, enhancement shape, and annular enhancement. Specific CEUS analysis components included: enhancement kinetics (categorized as fast wash-in [tumor enhancement faster than renal cortex], slow wash-in [tumor enhancement slower than cortex], or isoenhancing [tumor and cortex enhance simultaneously]); wash-out timing (fast wash-out [tumor washout faster than cortex], slow wash-out [tumor washout slower than cortex], or isoregressive [tumor and cortex washout concurrently]); peak intensity (high [tumor enhancement higher than cortex], isoenhancing, or low enhancement [tumor enhancement lower than cortex] relative to the renal cortex); post-enhancement morphology (regular or irregular); and enhancement homogeneity (uniform or heterogeneous). Pseudocapsule ring hyperenhancement indicated the presence of a circular hyperenhancement zone around the tumor, with significantly higher enhancement than the normal renal cortex inside and around the tumor.

### Quantitative analysis of CEUS images

Dynamic storage images in DICOM format were obtained and analyzed using Vuebox software. A region of interest (ROI) was delineated, with ROI1 representing the total area for image analysis, ROI2 indicating the area of uniform enhancement (in cases of inhomogeneous enhancement, the region with the highest enhancement intensity was selected while avoiding annular enhancement), and ROI3 encompassing the normal renal cortex with uniform enhancement at the same depth. Various time-intensity curve (TIC) parameters were measured, including (1) peak enhancement (PE), representing the intensity of peak enhancement; (2) rise time (RT); (3) mean transit time local (mTTI); (4) time to peak (TTP), signifying the time when the contrast intensity within the mass reached its peak enhancement; (5) fall time (FT); (6) wash in area under the curve (WiAUC), indicating the area under the curve from arrival time to peak enhancement; (7) wash in rate (WiR), defined as the tangent of the rising part of the TIC curve; (8) wash in perfusion index (WiPI), calculated as the ratio of WiAUC to time; (9) wash out rate (WoR), denoting the tangent of the descending part of the TIC curve; (10) wash out area under the curve (WoAUC); (11) wash in and wash out area under the curve (WiWoAUC); and (12) QOF.

### Statistical methods

Statistical analysis was performed using SPSS 23.0 statistical software. Age and tumor diameter were assessed for normal distribution and expressed as mean ± s.d. TIC parameters were expressed as median (interquartile range, IQR). Differences in TIC parameters between the lesions and normal renal cortex were compared using the paired Wilcoxon signed-rank sum test for paired samples. Statistical significance was determined by a *p*-value < 0.05.

## Results

### Patients and US results

**Fig. 1:**
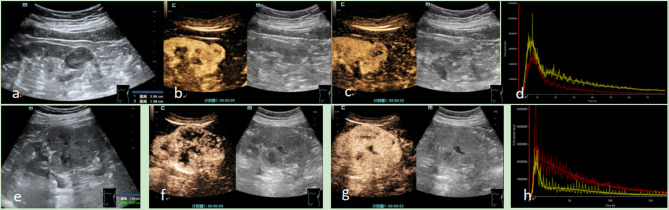
Representative CEUS images of nccRCC versus ccRCC. (**a-d**): A patient, female, 63 years old, with left chRCC. (**a**): Conventional ultrasound image displaying a hypoechoic mass in the left lower kidney, measuring 2.86 cm x 1.98 cm, with clear boundaries and a regular shape; (**b**): Contrast-enhanced ultrasound (CEUS) image during the perfusion phase at 9 s, demonstrating homogeneous low enhancement within the lesion and peripheral annular enhancement; (**c**): CEUS image during the washout phase at 52 s, depicting low enhancement within the lesion and peripheral ring enhancement; (**d**): Time-intensity curve (TIC) of nccRCC showing slow advance, fast retreat and low enhancement. (**e-h**): A patient, female, 72 years old, with left ccRCC. (**e**): Conventional ultrasound image displaying a hypoechoic mass in the left middle kidney, measuring 7.55 cm x 5.43 cm, with clear boundaries and a regular shape; (**f**): Contrast-enhanced ultrasound (CEUS) image during the perfusion phase at 9 s, demonstrating Heterogeneous high enhancement within the lesion and peripheral annular enhancement; (**g**): CEUS image during the washout phase at 52 s, depicting high enhancement within the lesion and peripheral ring enhancement; (**h**): Time-intensity curve (TIC) of nccRCC showing fast advance, slow retreat and high enhancement

In terms of general patient characteristics and conventional ultrasonography findings, all 21 tumor cases were solitary, comprising lesions located in the left kidney (*n* = 10), the right kidney (*n* = 11), the superior pole (*n* = 6), the inferior pole (*n* = 5), and the middle pole (*n* = 10) of the kidney, and the mean lesion diameter measured (3.55 ± 1.43) cm. The included patients exhibited male predominance (*n* = 16, 76.2%) with a median age of 55 years. Conventional ultrasound observations revealed predominant features such as hypoechoic appearance (10/21, 47.6%), absence of liquefaction (18/21, 66.7%), regular morphology (19/21, 90.5%), clear boundaries (21/21, 100%), absence of calcification (17/21, 81%), and limited color Doppler flow (only 1 case with grade III blood flow) (Fig. 1; Table 1). All cases were later confirmed as nccRCC through surgical and pathological evaluation at our hospital. This included cases of pRCC (*n* = 8, comprising 4 type I and 4 type II), chRCC (*n* = 5), renal cell carcinoma associated with Xp11.2 translocation/TFE-3 gene fusion (*n* = 4), sarcomatoid carcinoma (*n* = 1), collecting duct carcinoma (*n* = 1), and eosinophilic papillary carcinoma (*n* = 2).

**Table 1:** Clinical data, conventional ultrasound and CDFI indicators of nccRCC

Gender, *n*(%)		Echogenicity, *n*(%)	
Male	16(76.2%)	Hypo-	10(47.6%)
Female	5(23.8%)	Iso-	6(28.6%)
Age(years): mean ± STD	55.29 ± 11.89	Hyper-	5(23.8%)
Size(cm): mean ± STD	3.55 ± 1.43	Boundary, *n*(%)	
Subtypes of non-clear cell renal cell carcinoman, *n*(%)		Well defined	21(100.0%)
papillary renal cell carcinoma	8(38.1%)	Poorly defined	0(0.0%)
chromophobe renal cell carcinoma	5(23.80%)	Shape, *n*(%)	
Xp11.2 translocation / TFE-3 gene fusion related renal cell carcinoma	4(19.0%)	Regular	19(90.5%)
Sarcomatoid carcinoma	1(4.8%)	Irregular	2(9.5%)
collecting duct cancer	1(4.8%)	Calcification, *n*(%)	
Eosinophilic papillary carcinoma	2(9.5%)	Yes	4(19.0%)
Laterality, *n*(%)		No	17(81%)
Left	9(42.9%)	CDFI, *n*(%)	
Right	12(57.1%)	Grade 0	4(19.0%)
Location, *n*(%)		Grade I	14(66.7%)
Superior	6(28.6%)	Grade II	2(9.5%)
Middle	10(47.6%)	Grade III	1(4.8%)
Inferior	5(23.8%)		
cystic degeneration, *n*(%)			
Yes	3(33.3%)		
No	18(66.7%)		

### Results of qualitative analysis of CUES images

Qualitative analysis of ultrasonography findings in the 21 nccRCC patients demonstrated that the enhancement patterns included slow enhancement (16/21, 76.1%), synchronous enhancement (4/21, 19.0%), and rapid enhancement (1/21, 4.9%). Peak intensity observations revealed low enhancement (15/21, 71.5%), moderate enhancement (4/21, 19.0%), and high enhancement (2/21, 9.5%). Enhancement uniformity analysis indicated homogeneous enhancement (13/21, 61.9%) and inhomogeneous enhancement (8/21, 38.1%). The rapid decline in enhancement was observed in 8 cases (8/21, 38.1%), with a synchronous decline in 1 case (4.9%). Enhancement direction analysis showed diffuse enhancement (14/21, 66.7%) and concentric enhancement (7/21, 33.3%). Pseudocapsule ring hyperenhancement was present in 13 cases (13/21, 61.9%). Post-enhancement, the boundary appeared clear in 19 cases (19/21, 90.5%) and unclear in 2 cases (2/21, 9.5%). The post-enhancement range correlated with the grayscale ultrasound findings in 19 cases (19/21, 90.5%) and was enlarged in 2 cases (2/21, 9.5%), with one case being Type II pRCC and the other case being sarcomatoid carcinoma. The main characteristics observed in nccRCC patients included slow enhancement, rapid wash-out, low uniformity enhancement, and pseudocapsule ring hyperenhancement (Fig. 1; Table 2).

**Table 2:** Qualitative analysis results of CEUS in nccRCC, *n*(%)

Wash-in		Pseudocapsule ring hyperenhancement	
Fast	1(4.9%)	Yes	13(61.9%)
Synchronous	4(19.0%)	No	8(38.1%)
Slow	16(76.1%)	Enhancement direction	
Enhancement intensity		concentric	7(33.3%)
Enhancement	2(9.5%)	Eccentric	0(0%)
Homogeneous	4(19.0%)	diffuse	14(66.7%)
Heterogeneous	15(71.5%)	Enhanced shape	
Enhancement uniformity		regular	19(90.5%)
Uniform	13(61.9%)	irregular	2(9.5%)
Inhomogeneous	8(38.1%)	Enhanced range	
Wash-out		Expand	2(9.5%)
Fast	12(57.0%)	equate	19(90.5%)
Synchronous	1(4.9%)	shrink	0(0%)
Slow	8(38.1%)		

### Results of quantitative analysis of CUES images

In this study, a total of 21 nccRCC patients were included. Vuebox quantitative analysis software was employed to analyze the time-intensity curve of the lesions and normal renal parenchyma. TIC parameters were initially assessed using box plots, followed by the removal of outliers and supplementation through multiple random methods. Differences in TIC parameters between the lesions and normal renal cortex were evaluated using the Wilcoxon symbolic rank sum test for paired samples. The results indicated that PE, WiAUC, mTTI, WiR, WiPI, WoAUC, WiWoAUC, and WOR in the lesions were significantly lower than those in the normal renal cortex (Z = -3.980, -3.563, -2.427, -3.389, -3.980, -3.493, -3.528, -2.763, *P* < 0.001, < 0.001, = 0.015, = 0.001, < 0.001, < 0.001, < 0.001, = 0.006). However, there were no significant differences in RT, TTP, FT, and QOF between the lesions and normal renal cortex (Table 3).

**Table 3:** Quantitative analysis results of CEUS TIC parameters compared between lesions and normal renal cortex in nccRCC (M(Q_R_))

Parameters	Lesions	Normal renal cortex	Z	*P*
PE(a.u)	4.1382*10^10^(66348.06,1.4242*10^11^)	9.2941*10^10^(123555.47,3.7906*10^11^)	-3.980	< 0.001
WiAUC(a.u)	5.4502*10^10^(348755.98,4.8972*10^11^)	1.2585*10^11^(670435.66,1.4572*10^12^)	-3.563	< 0.001
RT(s)	4.830(4.650,7.920)	5.910(4.545,7.905)	-0.574	0.566
mTTI(s)	17.880(9.860,38.320)	30.030(13.435,58.500)	-2.427	0.015
TTP(s)	10.680(8.820,14.220)	11.620(8.840,13.200)	-1.025	0.305
WiR(a.u)	1.0491*10^10^(16712.79,3.8697*10^10^)	2.6576*10^10^(26210.92,1.0758*10^11^)	-3.389	0.001
WiPI(a.u)	2.5617*10^10^(41690.74,8.7887*10^10^)	5.8039*10^10^(79940.68,2.3537*10^11^)	-3.980	< 0.001
WoAUC(a.u)	6.3431*10^10^(654598.03,6.9036*10^11^)	1.481*10^11^(2.3871*106,2.607*10^12^)	-3.493	< 0.001
WiWoAUC(a.u)	1.1793*10^11^(940944.88,1.1801*10^12^)	2.7403*10^11^(3.0576*106,4.1815*10^12^)	-3.528	< 0.001
FT(s)	9.570(7.125,13.595)	10.830(6.810,18.755)	-1.443	0.149
WOR(a.u)	5.6413*10^9^(10243.19,2.3964*10^10^)	8.308*10^9^(9022.63,5.5804*10^10^)	-2.763	0.006
QOF(%)	76.890(73.770,86.665)	78.480(75.390,89.725)	-1.095	0.274

PE = peak enhancement, RT = rise time, mTTI = mean transit time local, TTP = time to peak, FT = fall time, WiAUC = wash in area under the curve, WiR = wash in rate, WiPI = wash in perfusion index, WoR = wash out rate, WoAUC = wash out area under the curve, WiWoAUC = wash in and wash out area under the curve, QOF = quality of fit

## Discussion

It is widely acknowledged that the histopathological variations within renal cell carcinoma significantly impact prognosis and tumor biology. The 5-year survival rate for ccRCC stands at only 55–60%, whereas pRCC and chRCC exhibit significantly higher rates of 80–90% [10]. Consequently, the preoperative classification of renal cell carcinoma holds substantial importance for clinical management. CEUS offers real-time and dynamic insight into microperfusion within lesions, aiding in the differentiation of malignant and benign renal lesions and enhancing the evaluation of complex renal cysts [11]. According to the literature, most ccRCC cases typically present with fast enhancement and high peak intensity, which correlates with invasiveness [12]. However, there is limited literature on the quantitative analysis of CEUS in nccRCC. This study sought to elucidate the CEUS characteristics of 21 nccRCC cases, conducting both qualitative and quantitative analyses to open new avenues for the diagnosis of this disease.

Prior studies [13] have explored CEUS characteristics in nccRCC, emphasizing that nccRCC predominantly exhibits low enhancement in peak intensity and a contrast pattern characterized by slow advancement, rapid washout, and low enhancement. In this study of 21 nccRCC patients, “slow advancement” accounted for 76.19%, low enhancement for 71.43%, and “rapid washout” for 57.14% of cases. Enhanced uniformity was observed in 61.90% of cases, with diffuse enhancement in 66.67%. This angiographic pattern, primarily displaying “slow advancement, rapid washout, and diffuse homogeneous low enhancement”, is consistent with previous research findings [14]. The primary reason behind this “slow advancement, low enhancement” pattern may be attributed to nccRCC’s typically hypovascular nature, with fewer vascular components and greater interstitial content, resulting in slow progression and limited enhancement. The “rapid washout” phenomenon may be linked to the presence of pRCC and chRCC within this nccRCC subgroup, accounting for 61.9%. These tumor types often possess an incomplete capillary network, arteriovenous fistulas, and direct arterial-to-venous blood flow, leading to faster contrast medium clearance than in the surrounding renal cortex. Additionally, nccRCC tumors tend to grow slowly, with rare occurrences of necrosis and cystic degeneration. Consequently, they appear more homogeneous when reaching their peak enhancement. Literature reports [15] suggest that the pseudocapsule results from the deposition, ischemia, or necrosis of fibrous tissue in the adjacent renal tissue during tumor growth, with circular enhancement being a common CEUS manifestation of the pseudocapsule. The contrast-enhanced pseudocapsule sign has demonstrated utility in differentiating benign and malignant renal tumors, with an AUC of 0.777 (95% confidence interval 0.701–0.853), sensitivity of 67.4%, and specificity of 88.0% [16]. However, multivariate Logistic regression analysis showed that the pseudocapsule sign is an independent predictor of RCC [16]. Zhu et al. [17] reported a significant difference in the incidence of CEUS pseudocapsule visualization among RCC patients across various tumor-size subgroups. Notably, the highest pseudocapsule visualization rate was observed in medium-sized tumors (with a diameter of 2–4 cm), reaching 79.3%. Furthermore, there was a statistical difference in the detection rate of CEUS pseudocapsules between ccRCC and nccRCC. Specifically, the detection rate of pseudocapsules in pRCC and chRCC was higher than that in ccRCC, possibly due to the distinct contrast enhancement patterns observed in different subtypes of renal cell carcinoma. pRCC and chRCC are categorized as hypovascular lesions, displaying slow progression and limited enhancement during perfusion in CEUS, followed by rapid fading. Consequently, high-echo rings could be distinctly visualized surrounding the tumor [18, 19]. However, multivariate analysis indicated that the pseudocapsule ring enhancement index is not an independent predictor for distinguishing RCC subtypes [15]. The abovementioned CEUS characteristics primarily rely on subjective qualitative analysis, are susceptible to human factors, and are characterized by subjectivity and low reproducibility.

Vuebox introduces a novel approach for quantitatively evaluating CEUS characteristics. In our study, we employed the quality of fit to assess curve fitting reliability. There were no significant differences in QOF between lesions and the normal renal cortex, and all curves had a GOF > 0.7, indicating reliable and comparable results. Time-related parameters such as RT, TTP, and FT represent the speed and quantity of contrast agent in the flushing stage, reflecting neovascularization within the mass [20]. In our study, RT, TTP, and FT for lesions were lower than those for the normal cortex, although without significant differences (*P* < 0.05), which might be attributed to the kidney’s single blood supply, with the tumor relying on the renal artery or accessory renal artery, similar to the normal renal cortex, resulting in no significant difference in blood flow perfusion rate. However, the perfusion time parameter mTTI within the lesion was significantly lower than that within the normal renal cortex (*P* < 0.05), suggesting a shorter overall perfusion time within the lesion, possibly due to the diminished blood supply in nccRCC. PE, WiAUC, WoAUC, and WiWoAUC parameters reflect microcirculation within the tumor. WiAUC, WoAUC, and WiWoAUC represent the time-intensity integral during inflow, outflow, and the combined inflow and outflow phases, respectively, offering a comprehensive and intuitive view of microvessel density within renal tumors and serving as unique quantitative Vuebox indices [21]. Our study results revealed that PE, WiAUC, WoAUC, and WiWoAUC were significantly lower within the lesion than in the normal renal cortex, indicating reduced perfusion, washout, and global blood flow within the lesion. To mitigate potential interference from PE and WiAUC caused by technical or individual variability, we further evaluated relative indicators such as WiPI, WiR, and WoR, ensuring the independence of curve parameters. Our findings showed that WiPI, WiR, and WoR within the lesions were lower than those in the normal renal cortex, with statistically significant differences. This implies reduced speed and quantity of contrast media during perfusion and washout phases within the lesion, leading to low enhancement. These results align with the qualitative analysis findings discussed earlier.

This study qualitatively and quantitatively analyzed the CEUS characteristics of nccRCC, improved the understanding of the disease, and provided a basis for clinical differential diagnosis. Ping Zhao et al. [13] showed that CEUS and contrast-enhanced magnetic resonance imaging (MRI) showed good diagnostic performance in the differential diagnosis of ccRCC and non-ccRCC, with AUC of 0.834 and 0.803 respectively, and there was no significant difference between the two methods (*p* = 0.54). Rong-xi Liang et al. [9] retrospectively analyzed CEUS and contrast-enhanced CT images of 82 cases with ccRCC, 24 cases with pRCC and 19 cases with ChRCC. The results showed that the enhancement patterns of CEUS and contrast-enhanced CT in the three subtypes of RCC were similar, and all of them could accurately diagnose the lesions of ccRCC, pRCC and ChRCC. Microbubble ultrasound contrast agent is a real blood pool imaging agent, which will not spread to the intercellular space, which greatly improves the sensitivity of blood flow detection at low flow rate and accurately reflects renal tumor perfusion, especially for nccRCC with lack of blood supply. Therefore, CEUS can be used as an alternative for patients with renal insufficiency or hypersensitivity to iodine contrast media that are not suitable for contrast-enhanced MRI or contrast-enhanced CT.

Indeed, the limitations of this study should be acknowledged. Firstly, this is a single-center retrospective study. Secondly, the inclusion of subjects who were pathologically confirmed after surgery may introduce selection bias. Thirdly, the sample size is relatively small, and the study did not differentiate between different subtypes of nccRCC, focusing mainly on the two most common subtypes. Additionally, rare subtypes were underrepresented in the study.

## Conclusion

In conclusion, when compared to ccRCC, nccRCC exhibits a higher 5-year survival rate. Thus, precise preoperative classification of RCC holds substantial clinical value in assessing prognosis. CEUS in nccRCC show cases distinctive characteristics, including slow advancement, rapid washout, low uniformity enhancement, and annular enhancement. These characteristic CEUS patterns are helpful in distinguishing nccRCC from ccRCC. CEUS can be utilized as an alternative examination for patients who are contraindicated for contrast-enhanced MRI or contrast-enhanced CT.

## Data Availability

Data is available upon reasonable request.
