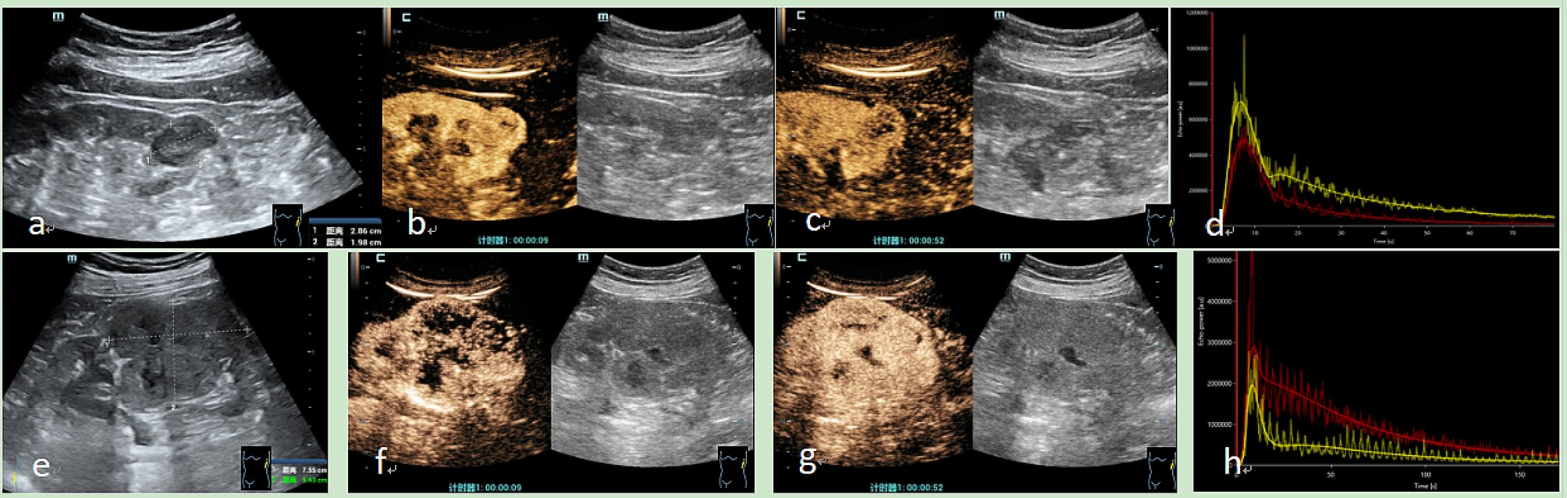# Correction: Qualitative and quantitative assessment of non-clear cell renal cell carcinoma using contrast-enhanced ultrasound

**DOI:** 10.1186/s12894-024-01527-3

**Published:** 2024-07-09

**Authors:** WeiPing Zhang, JingLing Wang, Li Chen, Jiayu Shi

**Affiliations:** 1 Department of Ultrasound, The First Affiliated Hospital, Jiangxi Medical College, Nanchang University, Nanchang, China; 2 The First Clinical Medical College, Jiangxi Medical College, Nanchang University, Nanchang, China

**Correction to: BMC Urology (2024) 24:129**

10.1186/s12894-024-01514-8

Following the publication of the original article [1], the wrong figure appeared as Fig. 1.; the figure should have appeared as shown below.

**Figure 1:**